# Characteristics of the Early Manifestations and the Sequential Changes of Magnetic Resonance Imaging in Acute Sheehan’s Syndrome: A Case Report and Literature Review

**DOI:** 10.7759/cureus.92275

**Published:** 2025-09-14

**Authors:** Aya Miura, Yasushi Kurihara, Kohei Kitada, Takuya Misugi, Daisuke Tachibana

**Affiliations:** 1 Department of Obstetrics and Gynecology, Osaka Metropolitan University Graduate School of Medicine, Osaka, JPN

**Keywords:** acute sheehan’s syndrome, hyponatremia, mri findings, pituitary atrophy, pituitary insufficiency, postpartum hemorrhage, sequential mri changes

## Abstract

Acute Sheehan’s syndrome (ASS) is a rare condition that causes pituitary insufficiency in the early postpartum period. In this article, we report a case of early diagnosis of ASS with hyponatremia as the initial symptom. In addition, we review the characteristics of the initial symptoms and sequential changes on magnetic resonance imaging (MRI) based on previous related cases. The patient was a 33-year-old pregnant woman, gravida 2, para 1. She had a vaginal delivery at 40 weeks of gestation, with a total blood loss of 12,700 mL due to atonic bleeding, and, subsequently, she developed a vulvar hematoma. She had a mild comatose, headache, and nausea 30 hours after delivery. The laboratory evaluation revealed hyponatremia (sodium: 128 mEq/L) and mild hypopituitarism (low adrenocorticotropic hormone and low cortisol). The patient was diagnosed with ASS and treated with hormone replacement therapy. MRI examination showed marginal enhancement of the pituitary gland at one week postpartum, normalization at three months, and atrophy at eight months. ASS should be considered when hyponatremia occurs after massive postpartum hemorrhage. It is also important to note that MRI findings in ASS can change during the postpartum period.

## Introduction

Sheehan’s syndrome is a rare condition, with a reported incidence of 1-3.1%, in which severe hypotension caused by massive hemorrhage leads to panhypopituitarism as a result of postpartum pituitary necrosis [1,2]. Sheehan’s syndrome usually presents with prolonged, nonspecific fatigue, lactational failure, and failure to resume menstrual cycles due to hypothyroidism and hypocortisolism [3]. Typical Sheehan’s syndrome develops gradually over several years after delivery, whereas cases that develop within days to weeks after delivery are referred to as acute Sheehan’s syndrome (ASS) [4]. ASS rapidly causes symptoms such as headaches, nausea, and general fatigue, but early diagnosis is difficult due to the variety of symptoms. Detecting and treating ASS as quickly as possible is recommended because long-term hormone replacement therapy is often required. Regarding diagnosis, magnetic resonance imaging (MRI) plays an important role by showing characteristic findings of the pituitary gland. However, these findings may vary depending on the timing, and few reports have documented the sequential MRI changes in detail.

We report a case of ASS diagnosed with hyponatremia on the second postpartum day and present the characteristics of the initial symptoms of ASS. Furthermore, we describe the sequential changes in MRI findings with a review of the literature.

## Case presentation

The patient was a 33-year-old woman (gravida 2, para 1) who conceived spontaneously and was transferred to our hospital due to massive postpartum hemorrhage. Her medical and family history were unremarkable. During her pregnancy, cervical cerclage was performed at 21 weeks of gestation for cervical incompetence, and the cerclage string was removed at 36 weeks of gestation. She delivered a healthy 2,530 g girl at 40 weeks of gestation. A cervical laceration extending to the fornix was sutured by the referring obstetrician. At that time, postpartum hemorrhage exceeded 2,000 mL. Subsequently, she developed a vulvar hematoma and required a blood transfusion; however, hypovolemic shock persisted, and the patient was transferred to our hospital. Upon arrival, she presented with clouded consciousness, a blood pressure of 60/40 mmHg, and a pulse rate of 150 beats/minute. Initial hematological data were hemoglobin of 4.9 g/dL, platelets of 75,000 /µL, and fibrinogen of 121 mg/dL. Laboratory tests confirmed the presence of disseminated intravascular coagulation (DIC). She received a large amount of fluids and blood transfusions due to hypovolemic shock and DIC. Angiography with obturator artery embolization using N-butyl-2-cyanoacrylate was performed for the vulvar hematoma with extravascular leakage [5]. During the treatment, she repeatedly exhibited shock vital signs. The total blood loss was 12,700 mL, and the total blood transfusion included 5,880 mL of red blood cells, 4,560 mL of fresh frozen plasma, and 1,000 mL of platelets.

Thirty hours after delivery, the patient developed an altered mental state (Glasgow Coma Scale score: 13 points), headache, and nausea. Laboratory evaluation revealed hyponatremia (sodium: 128 mEq/L, reference range: 138-145 mEq/L) and mild hypopituitarism (adrenocorticotropic hormone (ACTH): 11.3 pg/mL, reference range: 6.6-63.2 pg/mL; cortisol: 4.1 µg/dL, reference range: 3.7-19.4 µg/dL). Considering the postpartum massive hemorrhage and the laboratory findings, hypopituitarism due to ASS was suspected, and hormone replacement therapy (thyroxine and hydrocortisone) was initiated.

**Figure 1:**
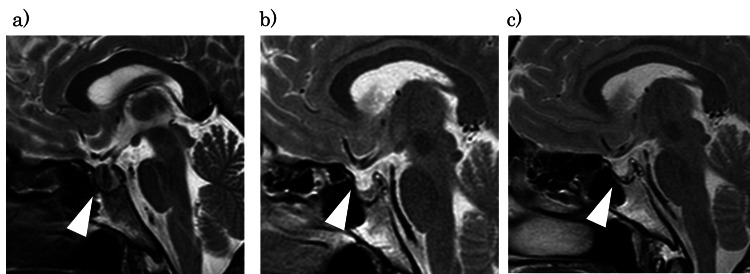
Sequential MRI changes of the pituitary gland in acute Sheehan’s syndrome. (a) Seven days postpartum: marginal enhancement. (b) 2 months: normalization. (c) 6 months: atrophy. The white arrowhead shows the pituitary gland.

On the seventh day postpartum, a head MRI showed a hook-shaped enhancement in which the absence of central enhancement of the pituitary gland was visualized without any internal contrast effect (Figure 1a). The hyponatremia improved with the administration of steroids and hypertonic saline, allowing a transition to oral medication, and the patient was discharged on the 12th postpartum day. At three months postpartum, MRI showed that her pituitary gland had returned to a normal size with contrast enhancement; however, steroid treatment was continued due to persistent impairment of hormone secretion (Figure 1b). In addition, estrogen and progesterone therapy were initiated because spontaneous lactation and menstruation were absent. At eight months postpartum, MRI revealed atrophy of the pituitary gland (Figure 1c), and hormone therapy was continued.

## Discussion

Sheehan’s syndrome generally occurs following postpartum hemorrhage, characterized by severe hypotension or hemorrhagic shock, and the condition affects 1-3.1 % of women who lose 1-2 L of blood with associated hypotension [2,6,7]. During pregnancy, the pituitary gland enlarges due to the proliferation of prolactin-secreting cells, resulting in diffuse nodular hyperplasia. Consequently, the pituitary gland becomes approximately twice as large compared to the non-pregnant state [8]. This pregnancy-induced pituitary enlargement may compress the superior hypophyseal artery against the sellar diaphragm, potentially causing mild ischemia [9]. Additionally, the pituitary’s vascular architecture makes it particularly susceptible to ischemia in cases of arterial hypotension or venous congestion. Sudden changes in arterial pressure during delivery, especially severe hypotension or shock following massive hemorrhage, can trigger arterial spasm in small vessels and pituitary apoplexy, ultimately resulting in infarction due to impaired blood flow to the anterior lobe of the pituitary gland [10]. In the present case, the patient experienced multiple episodes of hemorrhagic shock during the current delivery, which may have contributed to pituitary ischemia leading to ASS.

Although both typical Sheehan’s syndrome and ASS result from pituitary insufficiency, ASS is considered to present differently from typical Sheehan’s syndrome in terms of initial symptoms and MRI findings due to the rapid changes in circulatory dynamics. Few reports have addressed ASS, and the comprehensive characterization of early MRI findings and clinical manifestations is still limited. We identified 12 relevant reports of ASS involving MRI examinations (14 cases, including our case), which are summarized in Table 1 [2,4,10-19].

**Table 1:** Summary of the literature review findings for acute Sheehan’s syndrome. Hb = hemoglobin; ASS = acute Sheehan’s syndrome; MRI = magnetic resonance imaging; VD = vaginal delivery; CS = cesarean section; HRT = hormone replacement therapy; UAE = uterine embolization

Case (years)	Age	Delivery	Days	Cause of hemorrhage	Blood loss (mL)	Hb (g/dL)	First sign of ASS	Blood test	MRI findings			Treatment
									~2 weeks	2 weeks~3 months	3 months~	
Lavalle et al. (1995) [10]	30	VD	1	Uterine inversion	-	7.6	Headaches, convulsions	Hyponatremia, adrenal insufficiency	(6 D) Enlargement, peripheral enhancement	(1 M) normal size, noncontrast	-	HRT
Dejager et al. (1998) [11]	32	VD	3	Hypotension caused by epidural anesthesia	Little	-	Headaches, nausea, urination	Hypopituitarism	(6 D) Enlargement, no contrast enhancement	(1 M) Normal size	(3 M) Empty sella	HRT
Lust et al. (2001) [12]	32	VD	3	Atonic bleeding	3,200	-	Headaches	Hyponatremia, hypopituitarism	(5 D) Enlargement, hook-shaped enhancement	-	(4 M) Empty sella	HRT
Bunch et al. (2002) [13]	23	CS	6	Atonic bleeding	Massive	-	General fatigue	Hyponatremia, adrenal insufficiency	(6 D) Enlargement, hook-shaped enhancement	(1 M) Normal size	-	HRT
Munz et al. (2004) [14]	33	CS	6	Atonic bleeding	Massive	3	Headaches, vomiting	Hyponatremia, adrenal insufficiency	(6 D) No remarkable change	-	-	HRT
Wang et al. (2005) [15]	33	CS	19	Previa	Massive	6.6	Hemodynamic instability	Adrenal insufficiency	-	(19 D) Normal size	-	HRT
										(1M) mild atopy		
Kaplun et al. (2008) [16]	29	VD	17	Retained placenta	Massive	3.8	General fatigue, headaches	Hyponatremia, hypopituitarism	-	(26 D) Normal size	(6 M) Empty sella	UAE
Kaplun et al. (2008) [16]	21	VD	3	Perineal laceration	Massive	5.5	Fever, headache	Hyponatremia, adrenal insufficiency	(6 D) Hook-shaped enhancement	(5 W) Mild atopy	-	HRT
Anfuso et al. (2009) [17]	35	VD	8	-	500	8.8	General fatigue, headache	Hyponatremia, adrenal insufficiency	(8 D) Lack of enhancement	-	(3 M) Necrosis	HRT
Sasaki et al. (2014) [18]	37	VD	4	Retained placenta	3,600	4	-	Hyponatremia, hypopituitarism	(10 D) Enlargement, hook-shaped enhancement	-	(5 M) Atrophy	UAE HRT
Hale and Habib (2014) [19]	31	VD	6	Retained placenta	1,500	6.2	General fatigue, headaches	Hypopituitarism	(6 D) Enlargement, hook-shaped enhancement	-	-	HRT
Matsuzaki et al. (2017) [4]	27	VD	8	Atonic bleeding	5,000	4.1	Grand mal convulsion	Hyponatremia, adrenal insufficiency	-	(15 D) Normal size	(6 M) Atrophy	UAE, HRT
Pineyro et al. (2022) [2]	34	VD	7	Atonic bleeding	Massive	-	Headaches, comatose	Hyponatremia, hypothyroidism	(7 D) Enlargement, hook-shaped enhancement	-	(7 M) Smaller	HRT
This case	33	VD	2	Vulvovaginal hematoma	12,700	4.9	Headaches, comatose, nausea	Hyponatremia	(7 D) Hook-shaped enhancement	(2 M) Normal size	(6 M) Atrophy	IVR, HRT

In our case, clinical manifestations associated with hyponatremia appeared 30 hours after delivery. Other cases exhibited symptom onset 1-19 days postpartum (mean: approximately seven days), making our case the second-earliest onset. A few reports provided detailed descriptions of blood loss and hemoglobin levels; however, no clear correlation was found between these factors and the timing of symptom onset. In some cases, symptom onset was delayed despite massive hemorrhage and severe anemia, whereas in others, symptoms developed despite a blood loss of only 500 mL. We speculated that the massive hemorrhage in this case led to repeated episodes of hemorrhagic shock, thus resulting in an earlier onset of pituitary insufficiency compared to other cases. These findings suggest that the severity of pituitary insufficiency varies depending on the extent of hemorrhagic shock, which may influence the timing of symptom onset.

In typical Sheehan’s syndrome, hyponatremia has been reported in approximately 5% of cases, whereas hyponatremia was observed in 11 of 14 cases (78.6%) in reports of ASS [12]. This suggests that hyponatremia may be common in ASS than in typical Sheehan’s syndrome. Various mechanisms have been proposed to explain the development of hyponatremia. Hypothyroidism and glucocorticoid deficiency resulting from pituitary insufficiency decrease free-water clearance and lead to urine hypertonicity [20]. Additionally, hypopituitarism stimulates vasopressin secretion, which results in inappropriate antidiuretic hormone secretion [20]. These hormonal changes may have contributed to hyponatremia due to hemodilution.

In ASS, severe hypovolemic shock due to massive hemorrhage is often managed with substantial fluid resuscitation and blood transfusions. As a result, excessive fluid accumulates in the body, leading to marked hemodilution. Furthermore, pituitary insufficiency-associated hypothyroidism and adrenal insufficiency reduce free-water clearance, and concomitant increased vasopressin secretion further exacerbates hemodilution. Thus, in ASS, the combination of fluid overload from resuscitation and hormonal abnormalities synergistically promotes hyponatremia, making it more frequent than in typical Sheehan’s syndrome.

Table 1 reveals that the MRI examination of the pituitary gland may show different features depending on the stage of the disease. In Sheehan’s syndrome, the pituitary infarction occurs due to hypotension associated with massive bleeding during delivery, resulting in an enlarged pituitary without contrast enhancement in ASS [7]. Therefore, during the early period (up to two weeks), MRI reveals an enlarged pituitary gland with central hypointensity on T1-weighted images and hyperintensity on T2-weighted images. This enhancement is referred to as “hook-shaped enhancement.” From two weeks to three months, the pituitary gland returns to normal size, and the contrast effect on MRI improves. After that, the pituitary gland gradually shrinks, initially enlarging non-hemorrhagically, then progresses to atrophy, and eventually develops an empty sella. In this report, we present sequential changes in MRI images. Therefore, even if ASS is suspected based on hormonal abnormalities, an MRI scan performed between two weeks and three months may show normal pituitary findings, potentially leading to misjudgment. In such cases, a follow-up MRI should be performed to confirm the empty sella and establish the diagnosis of ASS.

## Conclusions

This report is among the first to highlight trends in early manifestations and sequential MRI changes in ASS. In this report, we provided a comprehensive examination of the endocrine and imaging assessment of a patient presenting in critical condition due to pituitary necrosis in the immediate postpartum period. In cases of massive bleeding during labor, if manifestations such as impaired consciousness, headaches, and nausea are present alongside hyponatremia, the possibility of ASS should be considered.
